# Timing and microbiological profile influence long-term outcomes after debridement, antibiotics, and implant retention (DAIR) in acute hip periprosthetic joint infection

**DOI:** 10.1007/s00402-026-06385-2

**Published:** 2026-06-23

**Authors:** Ernesto Muñoz-Mahamud, Juan Carlos Perdomo-Lizarraga, Andrés Combalia, Alfonso Alías, Adrià Serra, Jenaro Ángel Fernández-Valencia, Miguel Ángel Verdejo, Álex Soriano

**Affiliations:** 1 Departament de Cirurgia i Especialitats Medicoquirúrgiques,Facultat de Medicina i Ciències de la Salut, Universitat de Barcelona (UB), c. Casanova, 143, 08036 Barcelona, Spain; 2 Department of Orthopaedics and Trauma Surgery, Hospital Clínic of Barcelona, University of Barcelona, Barcelona, Spain; 3 Department of Infectious Diseases, Hospital Clínic of Barcelona, University of Barcelona, Barcelona, Spain; 4 Institut d’Investigacions Biomèdiques August Pi i Sunyer (IDIBAPS), Barcelona, Spain; 5 Departament de Medicina, Facultat de Medicina i Ciències de la Salut, Universitat de Barcelona (UB), c. Casanova, 143, 08036 Barcelona, Spain

**Keywords:** Periprosthetic joint infection, Total hip arthroplasty, Debridement and implant retention, Surgical timing, Treatment outcomes, Risk factors

## Abstract

**Introduction:**

The strategy of debridement, antibiotics, and implant retention (DAIR) represents one of the main therapeutic modalities for acute periprosthetic joint infection (PJI). However, reported outcomes remain highly variable, with success rates ranging from 16 to 92%. This study aimed to evaluate long-term treatment failure-free survival and identify risk factors for treatment failure in patients with acute hip PJI treated with DAIR using time-to-event analytical methods.

**Material and methods:**

This retrospective study evaluated the treatment failure rate in 115 patients treated with the DAIR strategy for acute PJI following primary or aseptic revision hip arthroplasty between 1999 and 2018. Potential predictors of treatment failure-free survival, including patient characteristics, infection microbiology, and surgical timing, were analyzed using univariate tests, Cox proportional hazards regression (hazard ratio [HR]), and Kaplan–Meier survival estimates.

**Results:**

Among the 115 patients included, 56 were women and 59 were men; 48 were aged 75 years or younger and 67 were older than 75 years. In 88 cases, the infection occurred after primary arthroplasty, whereas 27 followed aseptic revision surgery. The mean follow-up was 7.1 years (SD 4.4). At five years, treatment failure occurred in 30.4% of cases. Procedures performed within seven days of diagnosis showed lower failure rates than those after the first week following diagnosis (27.5% vs 53.8%, p = 0.05; HR = 2.09, 95% CI 0.91–4.79). Failure was more frequent after revision arthroplasty compared with primary arthroplasty (48.1% vs 25%, p = 0.02; HR = 2.15, 95% CI 1.08–4.27) and in polymicrobial infections compared with monomicrobial infections (47.8% vs 24.5%, p = 0.04; HR = 2.32, 95% CI 1.13–4.77). No other variables showed a significant association with failure risk.

**Conclusion:**

Revision arthroplasty and polymicrobial infections were associated with higher failure rates after DAIR. Lower failure rates were observed among patients treated within seven days of diagnosis, although this finding should be interpreted with caution and warrants further investigation.

## Introduction

Periprosthetic joint infection (PJI) is one of the most serious complications following hip arthroplasty, accounting for approximately 13% – 17% of all hip revision procedures [1, 2]. For acute PJI, the most standardized treatment strategy is debridement, antibiotics, and implant retention (DAIR), a surgical approach aimed at eradicating infection while preserving well-fixed prosthetic components [3]. The procedure involves removal of infected and devitalized tissues, exchange of modular parts, thorough intraoperative irrigation, and administration of pathogen-directed antibiotic therapy [4].

Indications for this approach include infections occurring within three months of prosthesis implantation, symptoms lasting less than three weeks, a stable and functional implant, well-preserved soft tissues without fistula formation, and infections caused by susceptible microorganisms [3, 5]. The success of DAIR depends on a complex interplay of factors, including patient characteristics, microbiological features such as pathogen type and antimicrobial resistance, and procedure-related variables such as surgical timing and debridement quality [6, 7]. However, many studies have focused on relatively short follow-up periods, typically around two years, which may limit insight into the longer-term performance of DAIR. Although some series report follow-up of up to five years [8], additional data on mid- to long-term outcomes remain warranted. Therefore, the aim of our study was to evaluate treatment failure-free survival at five years and to identify clinical, microbiological, and procedural predictors of treatment failure in a cohort of patients with acute hip PJI managed with DAIR.

## Methods

This retrospective study evaluated the treatment failure rate in 115 patients treated with the DAIR strategy for acute PJI following primary hip arthroplasty or aseptic revision hip arthroplasty between January 1999 and May 2018. Acute PJI was defined as the presence of infection-related clinical manifestations lasting no longer than three weeks and occurring within 12 weeks of the index arthroplasty. All infections included in the study were early postoperative infections; acute haematogenous infections were not included. For analysis purposes, PJI was classified as early acute when it occurred within the first four postoperative weeks, and as delayed acute when it developed between weeks five and twelve [3]. This subdivision was adopted to evaluate whether outcomes after DAIR differed according to the timing of infection onset within the early postoperative period and is supported by a previous analysis of the same cohort, which found no significant differences in treatment-failure free survival between these two groups [9]. Baseline demographic and clinical variables collected included age, sex, type of arthroplasty (primary or aseptic revision), intraoperative culture results, microbiological characteristics (monomicrobial vs polymicrobial and Gram-positive vs Gram-negative infections), and timing variables, including time from index arthroplasty to DAIR and time from PJI diagnosis to DAIR. Although infections following primary arthroplasty and aseptic revision arthroplasty may represent clinically distinct populations, both groups were included in the present cohort to evaluate overall outcomes after DAIR for acute hip PJI. To account for this heterogeneity, type of arthroplasty was specifically analysed in the survival models. Age was categorized into clinically relevant groups (≤ 75 and > 75 years) to facilitate interpretation and comparison between groups. The mean follow-up was 7.1 years (SD 4.4), with a range of 0–20.3 years. Four patients (3.5%) without evidence of treatment failure were lost to follow-up before five years. Among the included patients, 88 (76.5%) developed infection following a primary arthroplasty, whereas 27 (23.5%) presented infection after an aseptic revision procedure (Table 1).

**Table 1:** Univariate analysis of clinical and microbiological factors associated with treatment failure after DAIR for acute hip PJI. Hazard ratios (HR) with 95% confidence intervals (CI) were estimated using Cox regression. Significant p-values are shown in bold. Percentages are presented by column.

Variable	All patients (n = 115, 100%)		Success 80 (69.6%)		Failure 35 (30.4%)		p-value	HR (IC 95%); p-value
	n	%	n	%	n	%		
Age								
≤ 75	67	58.3	48	60.0	19	54.3	0.56	1.28 (0.66–2.49);
> 75	48	41.7	32	40.0	16	45.7		0.46
Sex								
Female	56	48.7	35	43.8	21	60.0	0.10	1.70 (0.86–3.35);
Male	59	51.3	45	56.2	14	40.0		0.12
ASA								
I	11	9.6	8	10.0	3	8.5	0.87	0.77 (0.33–1.78);
II	48	41.7	36	45.0	12	34.3		0.53
III-IV	33	28.7	23	28.8	10	28.6		
N/A	23	20.0	13	16.2	10	28.6		
Type of Arthroplasty								
Primary	88	76.5	66	82.5	22	62.9	**0.02**	2.15 (1.08–4.27);
Aseptic revision	27	23.5	14	17.5	13	37.1		**0.02**
Intraoperative culture results								
Confirmatory	72	62.6	49	61.3	23	65.7	0.64	1.16 (0.58–2.34);
Non-confirmatory	43	37.4	31	38.7	12	34.3		0.66
Type of infection								
Monomicrobial	49	42.6	37	46.3	12	34.3	**0.04**	2.32 (1.13–4.77);
Polymicrobial	23	20	12	15.0	11	31.4		**0.02**
Type of organism								
Gram +	44	38.3	32	40.0	12	34.3	0.28	0.82 (0.40–1.69);
Gram -	28	24.3	17	21.3	11	31.4		0.59
Time from index arthroplasty to DAIR								
≤ 4 weeks	91	79.1	62	77.5	29	82.8	0.51	0.82 (0.34–1.97);
5—12 weeks	24	20.9	18	22.5	6	17.1		0.65
Time from PJI diagnosis to DAIR								
≤ 7 days	102	88.7	74	92.5	28	80.0	**0.05**	2.09 (0.91–4.79);
> 7 days	13	11.3	6	7.5	7	20.0		0.08
≤ 2 days	65	56.5	46	57.5	19	54.3	0.74	1.10 (0.56–2.13);
3—21 days	50	43.5	34	42.5	16	45.7		0.78

*N/A *not available

Separately, the interval between diagnosis and the DAIR procedure was analysed as a surgical timing variable. For this analysis, procedures performed within seven days of diagnosis were compared with those performed later. Delayed DAIR procedures were not scheduled according to a predefined protocol. The interval between diagnosis and surgery reflected real-world clinical practice, and in some cases surgery was performed later due to factors such as persistent wound drainage, initial conservative management, or temporary medical contraindications. The diagnosis of PJI was established according to the criteria of the 2018 International Consensus Meeting [9]. The DAIR procedure was considered in patients with stable implants and preserved soft tissue. DAIR procedures were performed through the previous surgical approach. Necrotic and devitalised tissues were extensively debrided, and the surgical field was irrigated with 9 L of sterile saline solution. Until 2008, irrigation was performed using a conventional low-pressure system, whereas pulsatile lavage was routinely adopted thereafter [10]. No local antiseptics or local antibiotics were used during the procedure. According to institutional protocol, modular components were exchanged whenever feasible, although this variable was not systematically recorded throughout the study period. Multiple intraoperative samples were obtained before irrigation for microbiological analysis. Following surgery, empirical intravenous antibiotic therapy was initiated and subsequently adjusted according to microbiological culture results and antimicrobial susceptibility testing. Definitive antimicrobial treatment and treatment duration were determined by infectious diseases specialists dedicated to bone and joint infections according to the clinical course and laboratory findings, with treatment duration generally ranging from 6 to 12 weeks.

The cutoff point for outcome assessment was set at 5 years of follow-up to ensure a consistent observation period across the cohort, although longer follow-up was available for some patients. Treatment failure, defined as the primary endpoint, included any of the following events: revision surgery of the prosthetic joint for any cause, additional debridement over 12 weeks after the initial DAIR procedure, the requirement for long-term suppressive antibiotic therapy (SAT), or PJI-related mortality [10]. Revision surgery for any cause was included as part of the study endpoint, as any reoperation following DAIR, whether septic or aseptic, was considered to reflect an unfavorable clinical outcome. In addition, early aseptic revision within the follow-up period may be multifactorial and could be influenced by prior PJI and DAIR. Patients who died from causes unrelated to PJI during follow-up were not considered treatment failures. Revision surgery was defined as any surgical procedure requiring the removal or replacement of prosthetic components. The indication for revision was classified as septic when the patient met the ICM criteria for PJI [11]; cases not meeting these criteria were classified as aseptic. Patient follow-up was conducted from the date of the DAIR procedure until the end of the observation period or the last available clinical contact. Follow-up data were obtained from institutional medical records, including clinical visits, complications, re-interventions, and outcomes related to the DAIR procedure.

For the analysis of intraoperative culture results, cases were classified as microbiologically confirmed when ≥ 2 positive intraoperative culture samples yielding the same microorganism were present, and as not microbiologically confirmed when cultures were negative or only one positive sample was obtained. To analyze the variables of monomicrobial versus polymicrobial infections, as well as Gram-positive versus Gram-negative infections, only patients with microbiologically confirmed cultures were evaluated.

### Statistical analysis

Continuous data are presented as mean ± standard deviation (SD), whereas categorical variables are expressed as frequencies and percentages. Continuous variables were categorised into clinically relevant groups for analysis, including age and timing variables as defined above. Group comparisons were performed using the Chi-square test. Cox proportional hazards regression models with 95% confidence intervals (CI) were applied to estimate the cumulative risk of failure. Clinically relevant variables, including age, sex, type of arthroplasty (primary vs aseptic revision), microbiological characteristics, and timing variables, were analysed using Cox regression models. To evaluate the potential influence of temporal changes in clinical practice, an additional analysis was performed comparing patients treated between 1999–2008 and 2009–2018. Kaplan–Meier analysis was used to evaluate treatment failure-free survival. Deaths unrelated to PJI were handled as simple censoring events at the time of death, as no competing risk model was applied. Data handling was conducted using Microsoft Excel, and all statistical analyses were performed with Jamovi software (version 2.6.44; The Jamovi Project, Sydney, Australia).

### Ethics statement

Patient information was obtained from hospital medical records. Data collection and analysis were conducted retrospectively following pseudo-anonymization procedures. The study protocol was approved by the Institutional Ethics Committee (reference HCB/2023–0492).

## Results

### Treatment failure-free survival

The five-year prosthetic joint revision rate was 27% (31/115), including 27 septic (87.1%) and 4 aseptic (12.9%) revisions. The overall five-year treatment failure rate was 30.4% (35/115), including 31 prosthetic revisions and 4 patients without prosthetic revision: 1 s DAIR performed > 12 weeks, 1 patient managed with SAT, and 2 infection-related deaths.

The overall mortality rate during the five-year follow-up period was 17.4% (20 patients). Eight patients (7%) underwent prosthetic revision prior to death, while twelve patients (10.4%) died without undergoing revision surgery. Among these, two deaths (1.7%) were related to infection, whereas the remaining ten patients (8.7%) died from other unrelated medical conditions. Infection control required suppressive antibiotic therapy (SAT) in 2 patients (1.7%), one of whom subsequently required prosthetic revision. Two patients (1.7%) underwent an additional debridement more than 12 weeks after the initial DAIR procedure, one of whom subsequently required prosthetic revision.

### Risk factors

The five-year treatment failure rate was 37.5% in females and 23.7% in males (p = 0.10), with no significant differences across age groups (≤ 75 years: 28.4% vs > 75 years: 33.3%; p = 0.56). Early acute PJI showed a five-year treatment failure rate of 31.9%, compared with 25% in delayed acute PJI (p = 0.51). However, the type of arthroplasty showed a significant impact on outcomes, with higher treatment failure rates after aseptic revision arthroplasty compared with primary arthroplasty (48.1% vs 25%; p = 0.02). Cox regression confirmed this association (HR 2.15; 95% CI, 1.08–4.27; p = 0.02), indicating that type of arthroplasty was significantly associated with treatment failure.

Patients with microbiologically confirmed intraoperative cultures showed a five-year failure rate of 31.9%, compared with 27.9% among those without microbiological confirmation (p = 0.64). Gram-negative infections tended to yield higher failure rates than Gram-positive infections (39.3% vs 27.3%; p = 0.28). Polymicrobial infections were associated with higher five-year failure rates than monomicrobial infections (47.8% vs 24.5%; p = 0.04). Cox regression showed a hazard ratio (HR) of 2.32 (95% CI, 1.13–4.77; p = 0.02).

DAIR performed within 7 days of diagnosis showed a lower five-year failure rate compared with procedures performed after the first week following diagnosis (27.5% vs 53.8%; p = 0.05). Cox regression analysis yielded a hazard ratio (HR) of 2.09 (95% CI, 0.91–4.79; p = 0.08), indicating that this association did not reach statistical significance. Patients who underwent DAIR within the first two days after diagnosis exhibited a five-year treatment failure rate of 29.2%, compared with 32% among those managed after two days, with no statistically significant difference (p = 0.59).

**Fig. 1:**
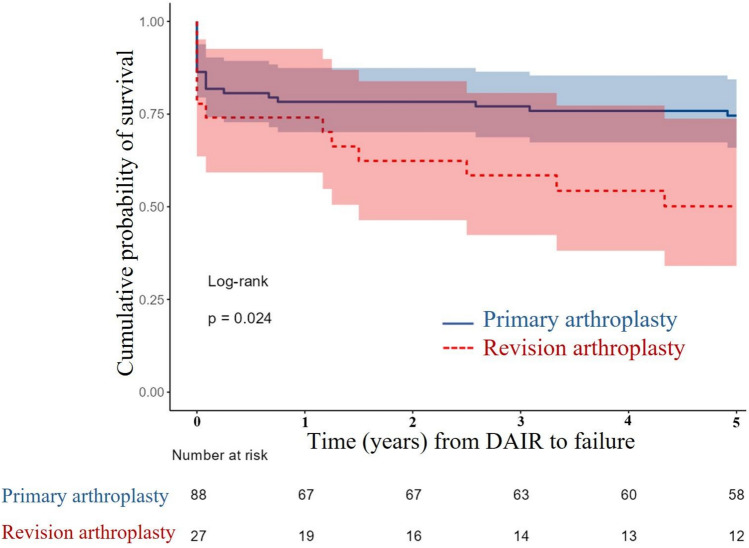
Kaplan –Meier curve comparing treatment failure-free survival between primary and revision arthroplasties

Patients treated between 1999–2008 (n = 47) and 2009–2018 (n = 68) showed similar treatment failure rates (29.8% vs 30.9%; p = 0.90). No significant association was observed between treatment period and treatment failure in Cox regression analysis (HR 1.08, 95% CI 0.55–2.13; p = 0.81), and Kaplan–Meier analysis demonstrated comparable treatment failure-free survival between both groups Fig. 1).

### Subgroup survival analyses

**Fig. 2:**
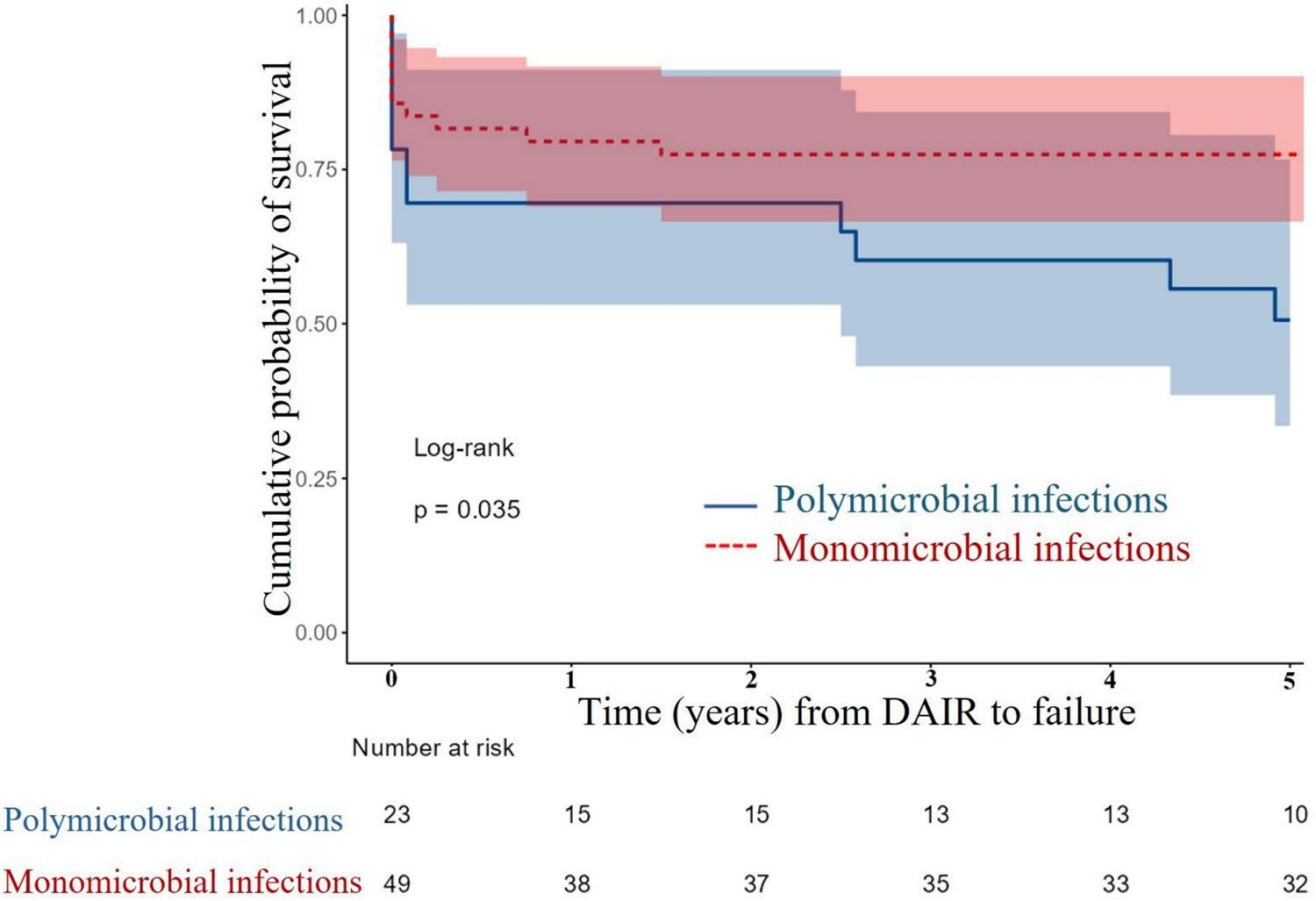
Kaplan–Meier curve illustrating reduced treatment failure-free survival in polymicrobial infections over 5 years

**Fig. 3:**
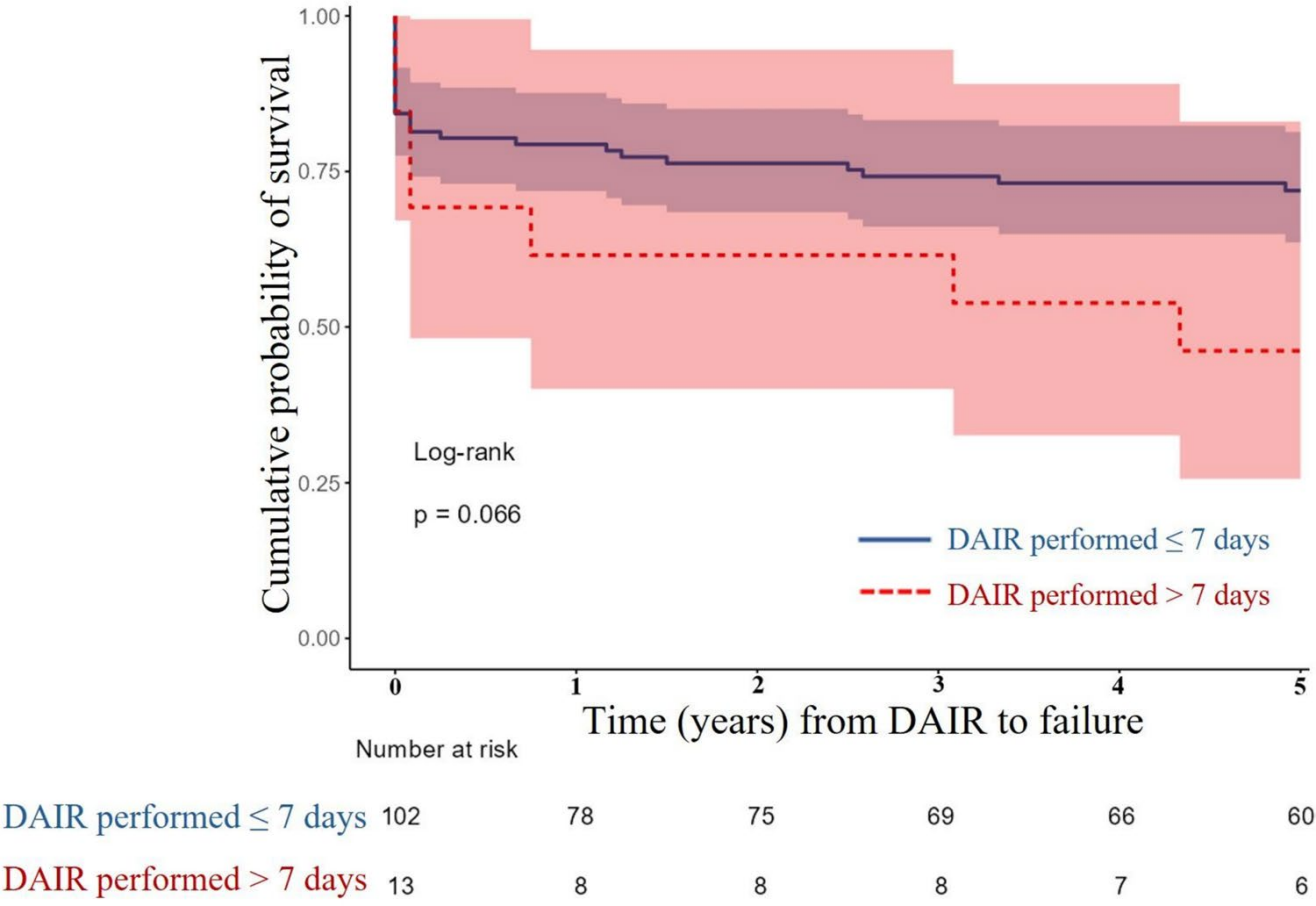
Kaplan–Meier curve showing treatment failure-free survival according to timing of DAIR (≤ 7 days vs 8–21 days)

**Fig. 4:**
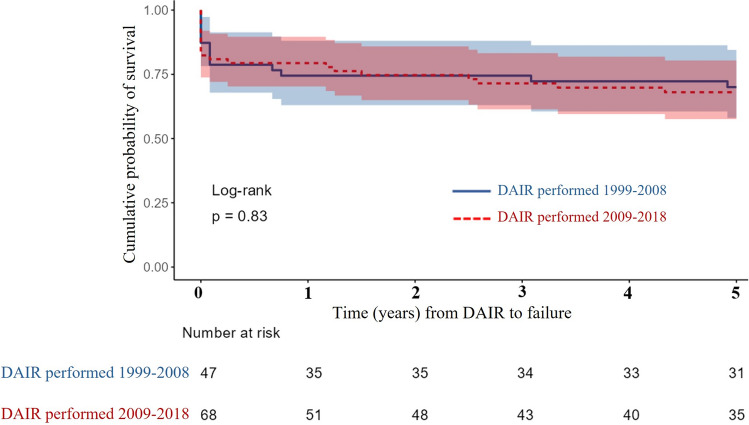
Kaplan–Meier curve showing treatment failure-free survival according to the period in which DAIR was performed (1999–2008 vs 2009–2018)

Kaplan–Meier analysis demonstrated lower five-year treatment failure-free survival in the revision group compared with primary arthroplasty (50.1% vs 74.6%; Fig. 2). Kaplan–Meier analysis similarly indicated lower five-year treatment failure-free survival in polymicrobial infections compared with monomicrobial infections (51.6% vs 77.4%; Fig. 3). Kaplan–Meier analysis demonstrated lower five-year treatment failure-free survival for DAIR performed after the first week following diagnosis compared with procedures performed within 7 days (46.2% vs 72%; Fig. 4).

## Discussion

The present study aimed to evaluate five-year outcomes after DAIR for acute hip PJI and to identify factors associated with treatment failure. The main findings were that polymicrobial infections and infections following aseptic revision arthroplasty were associated with higher failure rates, whereas lower failure rates were observed among patients treated within seven days of diagnosis, although this association did not reach statistical significance.

Outcomes following DAIR for acute PJI remain highly variable, reflecting differences in patient selection, microbiological characteristics, and surgical timing [7, 12–17]. The microbiological profile of infection is a well-recognized determinant of DAIR success. Chen et al. [18], in a series of 106 PJIs treated with DAIR, observed a failure rate of 20.6% in monomicrobial infections versus 55.6% in polymicrobial infections. Our results were consistent, with five-year failure rates of 24.5% and 47.8%, respectively. These findings support the hypothesis that polymicrobial infections are associated with more complex biofilm structures, broader antimicrobial resistance, and lower rates of eradication.

The impact of the type of arthroplasty on DAIR outcomes remains debated. Grammatopoulos et al. [17] analyzed 122 hip PJI cases treated with DAIR and found no significant difference in persistent infection between primary (31.7%) and revision arthroplasties (35%) (p = 0.7), with no association with implant survival (HR 0.8, 95% CI 0.3–2.2). In our cohort, we observed a clear disparity in five-year failure rates, with 25% in primary arthroplasties versus 48.1% in revision arthroplasties, indicating a substantially higher risk of failure after revision procedures. Infections following primary and aseptic revision arthroplasty may represent clinically distinct populations, with differences in surgical complexity and baseline risk. Accordingly, both groups were analysed within a single cohort to reflect routine clinical practice, and type of arthroplasty was specifically analysed in the survival models, where it was significantly associated with treatment failure. However, given the lack of comprehensive adjustment for baseline characteristics, these findings should be interpreted with caution.

The timing of surgical intervention is a key factor influencing DAIR outcomes. While guidelines differ regarding the acceptable interval from index arthroplasty [19], evidence suggests that the interval between diagnosis and debridement may be more critical. Löwik et al. [20] found no significant difference in failure rates between early and delayed acute infections, supporting the use of DAIR beyond four weeks. In contrast, Gupta et al. [21] reported higher success rates when DAIR was performed within one week of symptom onset. In our study, patients treated within one week of diagnosis showed lower five-year failure rates compared to those treated later (27.5% vs 53.8%). Shannon et al. [22] found no meaningful differences between very early time intervals, suggesting that urgent rather than emergent intervention may be appropriate. Accordingly, our study also did not demonstrate significant differences between procedures performed within two days and those performed later. Overall, the potential influence of surgical timing on DAIR outcomes warrants further investigation [4, 23].

This study has several limitations. First, its retrospective design may be associated with selection bias and incomplete data collection. Second, subgroup analyses reduced the number of patients in each group, potentially limiting statistical power. Third, deaths unrelated to PJI were handled as censoring events, and no competing risk analysis was performed. Fourth, baseline patient characteristics were not comprehensively adjusted for in the survival analyses, which may have affected the interpretation of some associations. Finally, important clinical variables such as host grade or comorbidity burden were not systematically available. In addition, although all included patients presented with symptoms lasting less than three weeks according to the study definition of acute PJI, the exact interval between symptom onset and DAIR was not systematically recorded and therefore could not be analysed. Likewise, although exchange of modular components was routinely performed according to institutional protocol, this variable was not systematically recorded throughout the study period and therefore could not be included in the analysis. Despite these limitations, the primary endpoint (treatment failure) is objective and clinically relevant. Moreover, the relatively long follow-up strengthens the reliability and clinical relevance of the findings. No significant differences were observed between the two study periods, suggesting limited influence of temporal changes in clinical practice. Larger multicentre studies are warranted to confirm these results.

In conclusion, lower five-year failure rates were observed among patients who underwent DAIR within seven days of diagnosis. However, this association did not reach statistical significance in Cox regression analysis and should therefore be interpreted with caution. Revision arthroplasty and polymicrobial infections were associated with higher failure rates. These findings suggest that microbiological characteristics may influence outcomes after DAIR, while the potential impact of surgical timing warrants further investigation.

## Data Availability

No datasets were generated or analysed during the current study.

## Declarations

### AI Tools use

GPT-3.5 (OpenAI, San Francisco, CA, USA) was used exclusively for language editing purposes.
